# Exploiting Machine Learning Algorithms and Methods for the Prediction of Agitated Delirium After Cardiac Surgery: Models Development and Validation Study

**DOI:** 10.2196/14993

**Published:** 2019-10-23

**Authors:** Hani Nabeel Mufti, Gregory Marshal Hirsch, Samina Raza Abidi, Syed Sibte Raza Abidi

**Affiliations:** 1 Division of Cardiac Surgery, Department of Cardiac Sciences, King Faisal Cardiac Center King Abdulaziz Medical City Ministry of National Guard Health Affairs - Western Region Jeddah Saudi Arabia; 2 College of Medicine-Jeddah King Saud bin Abdulaziz University for Health Ministry of National Guard Health Affairs Jeddah Saudi Arabia; 3 King Abdullah International Medical Research Center Jeddah Saudi Arabia; 4 Department of Surgery Faculty of Medicine Dalhousie University Halifax, NS Canada; 5 Department of Community Health and Epidemiology Faculty of Medicine Dalhousie University Halifax, NS Canada; 6 kNowledge Intensive Computing for Healthcare Enterprise Research Group Faculty of Computer Science Dalhousie University Halifax, NS Canada

**Keywords:** delirium, cardiac surgery, machine learning, predictive modeling

## Abstract

**Background:**

Delirium is a temporary mental disorder that occasionally affects patients undergoing surgery, especially cardiac surgery. It is strongly associated with major adverse events, which in turn leads to increased cost and poor outcomes (eg, need for nursing home due to cognitive impairment, stroke, and death). The ability to foresee patients at risk of delirium will guide the timely initiation of multimodal preventive interventions, which will aid in reducing the burden and negative consequences associated with delirium. Several studies have focused on the prediction of delirium. However, the number of studies in cardiac surgical patients that have used machine learning methods is very limited.

**Objective:**

This study aimed to explore the application of several machine learning predictive models that can pre-emptively predict delirium in patients undergoing cardiac surgery and compare their performance.

**Methods:**

We investigated a number of machine learning methods to develop models that can predict delirium after cardiac surgery. A clinical dataset comprising over 5000 actual patients who underwent cardiac surgery in a single center was used to develop the models using logistic regression, artificial neural networks (ANN), support vector machines (SVM), Bayesian belief networks (BBN), naïve Bayesian, random forest, and decision trees.

**Results:**

Only 507 out of 5584 patients (11.4%) developed delirium. We addressed the underlying class imbalance, using random undersampling, in the training dataset. The final prediction performance was validated on a separate test dataset. Owing to the target class imbalance, several measures were used to evaluate algorithm’s performance for the delirium class on the test dataset. Out of the selected algorithms, the SVM algorithm had the best F1 score for positive cases, kappa, and positive predictive value (40.2%, 29.3%, and 29.7%, respectively) with a *P*=.01, .03, .02, respectively. The ANN had the best receiver-operator area-under the curve (78.2%; *P*=.03). The BBN had the best precision-recall area-under the curve for detecting positive cases (30.4%; *P*=.03).

**Conclusions:**

Although delirium is inherently complex, preventive measures to mitigate its negative effect can be applied proactively if patients at risk are prospectively identified. Our results highlight 2 important points: (1) addressing class imbalance on the training dataset will augment machine learning model’s performance in identifying patients likely to develop postoperative delirium, and (2) as the prediction of postoperative delirium is difficult because it is multifactorial and has complex pathophysiology, applying machine learning methods (complex or simple) may improve the prediction by revealing hidden patterns, which will lead to cost reduction by prevention of complications and will optimize patients’ outcomes.

## Introduction

### Background

Delirium or acute confusion is a temporary mental disorder that occurs among hospitalized patients [1]. The Society of Thoracic Surgeons defines delirium as a mental disturbance marked by illness, confusion, and cerebral excitement, with a comparatively short course [2], developing over a short period (usually from hours to days) and which tends to fluctuate during the day [3]. Delirium symptoms range from a disturbance in consciousness (eg, coma) to cognitive disorders involving disorientation and hallucinations. Delirium has a wide range of presentations, from extremely dangerous agitation to depression-like isolation and, on the basis of its presentation, it has 3 distinct subclasses—that is, hyperactive, hypoactive, and mixed [4]. This diversity of possible presentations, along with its sudden onset and unpredictable course, makes early detection challenging. Royston and Cox state that “from the patient’s point of view, delirium and subsequent cognitive decline are among the most feared adverse events following surgery” [5]. The diversity of delirium’s presentation, along with its sudden onset and unpredictable course, makes its early detection difficult; however, the ability to predict delirium in patients can play a fundamental role in initiating preventive measures that can significantly improve outcomes.

Patients undergoing cardiac surgery are at higher risk of developing delirium [6-9]. Several studies demonstrated a negative association between postoperative delirium and an increased morbidity and mortality [7-10]. Of particular concern is the strong relationship between delirium and postoperative infections in cardiac surgery patients [7,9,11].

Given the undesirable consequences of delirium on surgical outcomes, it is deemed useful to predict the potential incidence of delirium in patients to pre-emptively administer and plan for therapeutic interventions to deal with delirium and in turn improve the surgical outcomes. Typically, predictive models for delirium use a range of clinical variables, applied to conventional statistical methods, mainly logistic regression (LR) [12-14]. The current predictive models for delirium generally present a simplified linear weighted representation of the statistical significance of the clinical variables toward the prediction of delirium [15].

However, we argue that the prediction of delirium is quite complex given the multiplicity of reasons and confounding factors contributing to the manifestation of delirium in patients. Data mining methods can be used to uncover underlying relationships between variables to develop predictive models that can categorize the patient population into ones that have the propensity to develop delirium versus those that are less likely to develop delirium. Sometimes, these relationships or patterns cannot be easily explained yet appear to be essential and have a significant contribution to the improvement of the predictive model’s performance, even if it is minimal (eg, a 0.01% improvement in a model’s performance means that for every 1000 patients, 1 extra life is saved or a complication is prevented or an accident is avoided).

Artificial intelligence in health care, particularly the use of machine learning methods, provides a purposeful opportunity to discover such underlying patterns and correlations by mining the data leading to the *learning* of data-driven prediction models. Machine learning models have been successfully applied in medical data [16-22] to solve a wide range of clinical issues, such as myocardial infarction [23], atrial fibrillation [24], trauma [25], breast cancer [26-28], Alzheimer [29-31], cardiac surgery [22,32], and others [20,21,33-35].

The main objective of this study was to develop predictive models to pre-emptively predict the manifestation of agitated delirium in patients after cardiac surgery. Although discovering underlying hidden patterns is interesting and can be done using the data mining methods used in this work, this was not our main objective as the pathophysiology of delirium is considered multifactorial and complex to start with. The rationale is that if we can identify based on preoperative clinical parameters which patients are likely to develop postoperative delirium, then clinicians can initiate preventive and therapeutic measures in a timely fashion, to mitigate the undesirable effects of delirium. Our approach for predictive modeling is to investigate machine learning methods to *learn* the prediction models using retrospective clinical data for around 5500 patients over a 7-year period who received cardiac surgery at Queen Elizabeth II Health Sciences Center (QEII HSC) in Halifax, Canada. In this paper, several machine learning models were explored, including artificial neural networks (ANN), Bayesian belief networks (BBN), decision trees (DT), naïve Bayesian (NB), LR, random forest (RF), and support vector machines (SVM).

### Related Work

Although the prevalence of postoperative delirium is low (10%-25%), it is associated with cognitive deterioration coupled with a set of complications in surgical patients. The complexity of delirium comes from its relation to multiple risk factors and the accompanying uncertainty of its pathophysiology [10,11,36]; this leads to challenges in pre-emptively identifying patients that are likely to develop postoperative delirium. Several authors have indicated that delirium is associated with adverse outcomes and advocate early recognition to ensure preventive measures can be applied in a timely and effective manner [3,7,9,10,13,14,37]. Some of the proposed preventive interventions that have been shown to reduce the incidence of delirium in high-risk patients include early mobilization and use of patient’s personal aids (reading glasses, hearing aid, etc) [38]. However, the pre-emptive identification of postoperative delirium is clinically challenging.

A structured PubMed search using the PubMed Advanced Search Builder with the structure (“delirium”) AND “predictive model”, will result in only 38 items. If we direct our attention to all the research published focusing on delirium and cardiac surgery, query structure (“delirium”) AND “cardiac surgery”, we will get 485 items. If we combine all the 3 terms, query structure ((“delirium”) AND “cardiac surgery”) AND “predictive model”, we will narrow the results down to 4 items.

In recognition of the importance of delirium within the cardiac surgical population, some have attempted to develop a predictive model. In this work, we decided to focus on articles that were published in English and focused on developing a predictive model for the prediction of delirium after cardiac surgery in adult patients. The initial search resulted in 38 articles. After reviewing the articles’ abstracts, we excluded articles that were not written in English, not about cardiac surgery patients, and in which no statistical model was developed. We ended up with 16 articles that were available for review. Multimedia Appendix 1 represents a summary of most relevant studies that attempted to develop a model for the prediction of delirium after cardiac surgery on adult patients.

For patients who underwent cardiac surgery, Afonso et al [12] conducted a prospective observational study on 112 consecutive adult cardiac surgical patients. Patients were evaluated twice daily for delirium using Richmond Agitation-Sedation Scale (RASS) and confusion assessment method for the intensive care unit (CAM-ICU), and the overall incidence of delirium was 34%. Increased age and the surgical procedure duration were found to be independently associated with postoperative delirium. Similarly, Bakker et al [13] prospectively enrolled 201 cardiac surgery patients aged 70 years and above. They found that a low Mini-Mental State Exam score and a higher preoperative creatinine were independent predictors of postoperative delirium [13]. Unfortunately, both of these models were based on a small sample size (<250 patients) and did not have a validation cohort.

Research in the use of machine learning–based prediction models to detect delirium is rather limited, especially for cardiac surgery. Kramer et al [39] developed predictive models using a large dataset comprising medical and geriatrics patients that had the diagnosis of delirium in their discharge code and a control group of randomly selected patients from the same period who did not develop delirium. The prediction models performed well with the highest performance achieved by the RF model (receiver operating characteristic-area under the curve [ROC-AUC]≈91%). Although they argue that their data were imbalanced, they used the ROC-AUC as their evaluation metric, which does not consider the class imbalance. Davoudi et al [40] applied 7 different machine learning methods on data extracted from the electronic health (eHealth) record of patients undergoing major surgery in a large tertiary medical center to predict delirium; they found an incidence of 3.1%. They were able to achieve a ROC-AUC ranging from 71% to 86%. Owing to the class imbalance secondary to the low incidence of delirium and to improve the model’s performance, they applied data-level manipulation using over- and undersampling, which did not result in a significant improvement (ROC-AUC ranging from 79% to 86%). Lee et al [41] published a nice systematic review and identified 3 high-quality ICU delirium risk prediction models: the Katznelson model, the original PRE-DELIRIC (PREdiction of DELIRium in ICu patients), and the international recalibrated PRE-DELIRIC model. All of these models used LR modeling as the primary technique for creating the predictive model. In the same paper by Lee et al [41], they externally validated these models on a prospective cohort of 600 adult patients that underwent cardiac surgery in a single institution. After updating, recalibrating, and applying decision curve analysis (DCA) to the models, they concluded that the recalibrated PRE-DELIRIC risk model is slightly more helpful. They argue that available models of predicting delirium after cardiac surgery have only modest accuracy. The current models are suboptimal for routine clinical use. Corradi et al [42] developed a predictive model using a large dataset (~78,000 patients) over 3 years in a single center using a good number of feature set (~128 variables). Their model had very good accuracy and the ROC-AUC ~90% on their test dataset. They used the CAM to detect delirium in the intensive care (CAM-ICU) and regular patient wards. Lee et al [41] conducted a systematic review in search for prediction models for delirium specifically designed for cardiac surgery patients. They found only 3 high-quality models and externally validated them on a local population of 600 patients. They used several metrics to evaluate the recalibrated models on the validation cohort (ROC-AUC, Hosmer–Lemeshow test, Nagelkerke’s R2, Brier score, and DCA). In their analysis, the recalibrated PRE-DELIRIC prediction model performed better when compared with the Katznelson model. However, based on the DCA and the expected net benefit of both models, there appears to be limited clinical utility of any of the models.

## Methods

### Data Sources and Study Population

This single-center retrospective cohort study included patients who underwent cardiac surgery at the QEII HSC in Halifax, Canada, between January 2006 and December 2012. Over those 7 years, 7209 patients underwent cardiac surgery. The Maritime Heart Center (MHC) registry was used to create the dataset. The MHC registry is a prospectively collected, detailed clinical database on all cardiac surgical cases performed at the MHC since March 1995 with more than 20,000 patients and 500 different variables. The final dataset included 5584 patients who met our inclusion criteria and were successfully discharged (home, other institution closer to home, nursing home, or rehabilitation facility).

Delirium in the acquired database is coded as a binary outcome (Yes/No) and is defined as short-lived mental disturbance marked by illusions, confusion, or cerebral excitement, requiring temporary medical and/or physical intervention or a consultation, or extending the patient’s hospital stay. Intraoperative management varied depending on the anesthetist preferences and the patient clinical status. Although most patients were managed in a systematic approach based on standard of care, in the ICU, CAM-ICU was used to trigger further investigations if delirium was suspected. If delirium had been suspected after transfer from the ICU, the diagnosis was confirmed using different diagnostic criteria and screening tools (eg, Mini-Mental State Exam and CAM).

Full ethics approval was obtained from the Capital Health Research Ethics Board, in keeping with the Tri-Council Policy Statement: Ethical Conduct for Research Involving Humans. Informed consent was waved by the ethics board as the study did not involve therapeutic interventions or potential risks to the involved subjects.

### Predictive Modeling: Methodology and Methods

Our aim was to develop a prediction model that can identify patients who are at risk of developing delirium after cardiac surgery. We investigated relevant machine learning methods, each with a specific learning algorithm to correlate the patient presurgery variables with a probabilistic determination of delirium as per the observations noted in the cardiac surgery dataset. The rationale for working with multiple machine learning methods was to determine the effectiveness of the different methods and then to select the best performing model that can be used in a clinical setting to predict postoperative delirium in new patients.

We pursued the standard data mining methodology comprising 6 steps as shown in Multimedia Appendix 2. These steps are as follows: (1) *data acquisition*: This step involved the procurement of the required dataset from the source (in this case from the MHC), while complying with data access and secondary data usage protocols; (2) *data preprocessing:* This step involved the cleaning of the dataset by removing incomplete records and next identifying the significant features/variables to develop the prediction models; (3) *modeling strategy set-up*: This step involved the formulation of the modeling strategy in terms of data partitioning into training dataset (N=4476; 80% of original) and test dataset (N=1117; 20% of original), data presentation during training, model evaluation criteria; (4) *class imbalance and training dataset class optimization:* This step was introduced to address the target class imbalance in the original dataset, so as to minimize the effect of the dominant class on the performance of the predictive models. We explored data level techniques, such as over- and undersampling, to address the class imbalance in the final training dataset, resulting in the final balanced training dataset (n=1014)*.* (5) *model learning:* This step involved setting up different model configurations—that is, setting up the model parameters for the candidate machine learning methods—and learning the models by presenting the preprocessed training data (step 2) as per the modeling strategy (step 3). As model learning is an exploratory exercise where different model configurations and multiple instantiations of the model are pursued to account for the probabilistic nature of machine learning methods and to avoid overfitting, 10-fold stratified cross-validation was used; and (6) *model evaluation:* In this step, the learnt models are evaluated (against the predefined criteria) for their effectiveness to predict delirium using the test data.

### Data Preprocessing and Variables Selection

Characteristics of patients who developed delirium postoperatively were compared with patients who did not. The mean and standard deviation were used for continuous variables that had a normal distribution and were compared using the 2-sided *t* test. Continuous variables that were not normally distributed were reported using the median and interquartile range and were compared using the Wilcoxon rank sum test. Categorical variables were reported as frequencies and percentages and were analyzed by ^2^ (Chi-square) or Fisher exact test as appropriate. The Kruskal-Wallis test was used for ordinal variables. Next, exploratory data analysis followed by univariate LR analysis was applied to isolate key perioperative variables with significant influence on postoperative agitated delirium.

All measures of significance are 2-tailed, and a *P* value <.05 was considered statistically significant. Statistical analysis and the assessment of model’s performance was conducted using the R-Software, version 3.1.0 (R Project for Statistical Computing) [43]. On the basis of univariate LR analysis, 22 variables were used to generate the machine learning–based predictive models.

The basic premise of any DT model is that it recursively split features based on the target variable’s purity. The ultimate goal of the algorithm is to optimize each split on maximizing the homogeneity of the grouping at each split (also known as purity) [44-46]. A node having multiple classes is impure, whereas a node having only 1 class is pure. One of the useful features of RF is its ability to identify relevant variables by assigning variable importance measure to the input variables [44-46]. Variable importance in RF can be measured using either misclassification error, Gini index, or cross-entropy. Most machine learning experts discourage the use of misclassification error in tree-based models because it is not differentiable and, hence, less amenable to numerical optimization [44,46]. In addition, cross-entropy and the Gini index are more sensitive to changes in the node probabilities than the misclassification rate. Both Gini index and cross-entropy apply probability to gauge the disorder of grouping by the target variable. However, they are a bit different, and the results can vary. The Gini index measures how often a randomly chosen element from the set would be incorrectly labeled, starting with the assumption that the node is impure (Gini index=1) and subtracting the probabilities of the target variable. If the node is composed of a single class (also known as pure), then the Gini index will be 0. On the other hand, cross-entropy is more computationally heavy because of the log in the equation. Instead of utilizing simple probabilities, this method takes the log of the probabilities (usually the log base 2; any log base can be used, but it has to be consistent for the sake of comparison between different tree-based models). The entropy equation uses logarithms because of many advantageous properties (mainly the additive property) that can be very beneficial in imbalanced class distributions and multiclass target variables [44,46]. A cross-entropy of 1 indicates a highly disorganized node (impure node), whereas a cross-entropy of 0 indicates a highly organized node (pure node).

In RF, each tree in the forest is grown fully (unpruned) using bootstrap samples of the original dataset, the out-of-bag (OOB) samples are used as test samples. A random subset of variables *k* from the original input variables space *K (where k<K)* is used at each node. On the basis of a specific measure (eg, mean decrease in impurity, Gini index, and mean decrease in accuracy), variables are selected, and the process is repeated to the end of the tree. The performance of each tree is computed over the corresponding OOB sample. For each variable, its importance is calculated as the mean relative decrease across the forest of trees performance when the observations of this variable in the OOB sample are randomly permuted. As the Waikato Environment for Knowledge Acquisition software (WEKA) was used in this work to develop the RF model, it applies the cross-entropy method as its default method for variables importance ranking.

### The Issue of Outcome Class Imbalance

In our dataset, the outcome class distribution is notably imbalanced (only 11.4% of patients developed delirium). Typically, classification algorithms tend to predict the majority class very well but perform poorly on the minority class due to 3 main reasons [47-49]: (1) the goal of minimizing the overall error (maximize accuracy), to which the minority class contributes very little; (2) algorithm’s assumption that classes are balanced; and (3) the assumption that impact of making an error is equal.

Several data manipulation techniques can be applied to reduce the impact of this class imbalance: at the data level (oversampling minority class or undersampling the majority class) or at the algorithm level (applying different costs to each class) [47-49]. Although data manipulation methods can improve a model’s performance, these methods do have some drawbacks [49]. At the data-level manipulation, oversampling tends to artificially increase the number of the minority class by creating modified copies; it tends to overfit the results to the training set and consequently is likely to poorly generalize. On the other hand, because undersampling discards some of the majority class observations, it essentially bears the risk of losing some potentially important hidden information. Algorithm level manipulation involves some trial and error and can be sensitive to training data changes.

In real life, class imbalance cannot be avoided as it is a result of the nature of the problem and domain (eg, natural disasters and patient death). In our dataset, oversampling led to overfitting on the training dataset with suboptimal generalization when applied to the imbalanced dataset. As postoperative delirium is linked with a wide range of complications (from a minor temporary confusion that totally resolves with no sequalae to the other extreme of sepsis and death), it is very hard to associate it to a specific cost. As such, given the intent of this study, we decided to apply random subsampling to balance the training dataset and have equal representation of outcome classes, thus optimizing the training dataset for the models. We used the *SpreadSubSample* filter in WEKA [46] to produce a random subsample by undersampling the majority class (which can be done by either specifying a ratio or the number of observations). In our case, we specified a ratio of 1:1. By doing so, the filter generates a new balanced dataset by decreasing the number of the majority class instances, which reduces the difference between the minority and the majority classes. Undersampling is considered an effective method for dealing with class imbalance [50]. In this approach, a subset of the majority class is used to learn the model. Many of the majority class examples are ignored; the training set becomes more balanced, which makes the training more efficient. The most common type of undersampling is random majority undersampling (RUS). In RUS, observations from the majority class are randomly removed. The final balanced training dataset (N=1014, 1:1 delirium) was used to develop the models.

### Training With 10-Fold Cross-Validation and Test Datasets

In predictive modeling, it is a common practice to separate the data into training and test dataset. In an effort to avoid overfitting and overestimating the model’s performance, the test dataset is only used to evaluate the performance of the prediction model [44,46,51,52]. The problem of evaluating the model on the training dataset is that it may exhibit high prediction ability (overfitting), yet it fails when asked to predict new observations. To address this issue, cross-validation is commonly used to (1) estimate the generalizability of an algorithm and (2) optimize the algorithm performance by adjusting the parameters [44,46,51-53]. We applied stratified 10-fold cross-validation on the balanced training dataset (50% delirium). The test dataset was preserved imbalanced to simulate the real clinical scenario and evaluate the behavior of different methods. Several metrics were used, that are immune to class imbalance, to appraise the final model’s performance on the test dataset [44,46,47,49,51,52].

## Results

### Development of Prediction Models: Experiments and Results

We investigated a range of relevant predictive modeling methods—that is, function-based models (LR, ANN, and SVM), Bayesian models (NB and BBN), and tree-based models (C4.5 DT and RF)—to generate 7 prediction models (all developed using the same balanced dataset). All models were generated and tested using the WEKA software, version 3.7.10 [54]. The setting of the prediction models and the optimization steps that were applied in this research are available in Multimedia Appendix 3. These predictive modeling algorithms were chosen based on 2 main reasons: (1) their noted effectiveness in solving medical-related classification problems and (2) a strong theoretical background that supports predictive modeling via data classification [11,16,19,20,22,23,25,30,31,39,46,52,55-63]. Experiments were conducted on a MacBook Pro (Apple Inc; 15-inch, 2017) with a 3.1-GHz Intel Core i7 processor and a 16 GB RAM 2133 MHz, running a MacOS High Sierra Version 10.13.

### General Patients’ Characteristics and Important Variables in the Dataset

Given the above definitions and procedures, agitated delirium was documented in 11.4% patients (n=661). The majority of patients were men (74%). Coronary artery bypass graft (CABG) was the most commonly performed procedure (67%). Almost 56% stayed in the ICU for 24 hours or less. Only 2% suffered a permanent stroke. Patients who developed postoperative agitated delirium were older and had a significantly higher incidence of comorbid diseases. A higher proportion of patients who developed agitated delirium underwent a combined procedure (CABG plus valve). The median stay in the cardiovascular intensive care unit in hours was 4 times higher for patients who developed agitated delirium postoperatively, compared with patients who did not (*P*<.001). Univariate analysis of in-hospital mortality did not show any statistical significance (in-hospital mortality: 4.1% vs 3.6%; *P*=.57; Table 1).

Univariate LR analysis of all pre-, intra-, and postoperative variables that can contribute to the development of delirium was performed using appropriate statistical tests in the R-Software. Univariate LR was applied on all candidate variables with a *P* value of less than .05 in univariate LR analysis to extract odds ratio (OR) with 95% CI generated for each candidate variable. The candidate variables were ranked based on the how low is the actual *P* value, the Akaike information criterion (lower is better), and impact of variable on postoperative delirium (signified by the OR). Then WEKA was used to generate variable importance using the RF model. WEKA applies the cross-entropy method to assess purity of the candidate variables with the RF algorithm as its default method, as it is more sensitive to class imbalance. Variables that appear higher at the trees are considered more relevant [44,51,52,63]. This is represented by the percentage of decrease of impurity (or increase of purity) of the final model based on adding this specific attribute. The number of times the candidate variable appeared in any location in all of the created tree models through the RF ensemble model process is also a criterion used in WEKA. The more times a variable is being selected in the RF creation process, the higher likelihood of it being important for the classification of the final target variable. This is also reflected in the decrease of impurity measure as the more decrease in impurity, the higher number of times that variable appears, which can imply its importance. Table 2 displays the importance of each input variable used in our RF model and its rank compared with the univariate LR analysis.

**Table 1:** Patient characteristics (N=4467).

Patient characteristics			Delirium		*P* value	
			No (n=3960)	Yes (n=507)		
**Preoperative characteristics**						
	**Age (years)**				**<.001**	
		Mean (SD)	66 (11)	72 (10)		
		Range	19-95	25-91		
	Male gender, n (%)		2942 (74.3)	386 (76.1)	.36	
	Hypertension, n (%)		2970 (75)	401 (79)	.04	
	Diabetes mellitus, n (%)		1426 (36)	223 (44)	<.001	
	Cerebrovascular disease, n (%)		436 (11)	112 (22)	<.001	
	Chronic obstructive pulmonary disease, n (%)		531 (13.4)	104 (20.5)	<.001	
	Frail, n (%)		238 (6)	49 (9.7)	.002	
	Ejection fraction <30%, n (%)		436 (11)	106 (21)	<.001	
	Preoperative atrial fibrillation, n (%)		424 (10.7)	102 (20.1)	<.001	
	EURO II^a^ score >5%, n (%)		717 (18.1)	231 (45.6)	<.001	
	**Urgency, n (%)**				**<.001**	
		Elective (admitted from home)	1901 (48)	198 (39)		
		Need surgery during hospitalization	1742 (44)	223 (44)		
		Urgent/emergent (life threatening)	317 (8)	91 (18)		
**Intraoperative characteristics, n (%)**						**<.001**
	**Procedure**					
		Coronary artery bypass graft	2744 (69.3)	291 (57.4)		
		Aortic valve replacement	622 (15.7)	93 (18.3)		
		Mitral valve surgery^b^	170 (4.3)	20 (4)		
		CABG+AVR^c^	325 (8.2)	79 (15.6)		
		CABG+MV^d^ surgery	51 (1.3)	3.4 (17)		
	Repeat sternotomy		230 (5.8)	59 (11.6)	<.001	
**In-hospital morbidity, n (%)**						**<.001**
	Reintubation		79 (2)	48 (9.5)		
	New postoperative atrial fibrillation		1247 (31.5)	217 (42.8)		
	Pneumonia		174 (4.4)	101 (20)		
	Sepsis		40 (1)	35 (6.9)		
	Deep sternal wound infection		24 (0.6)	15 (3)		
	Blood products transfusion within 48 hours from surgery		990 (25)	269 (53)		
	Length of stay after surgery <1 week		2257 (57)	66 (13)		
	Discharged home		3513 (88.7)	301 (59.4)		

^a^EURO II: European System for Cardiac Operative Risk Evaluation II.

^b^Mitral valve replacement or repair.

^c^CABG+AVR: coronary artery bypass graft + aortic valve replacement.

^d^CABG+MV: coronary artery bypass graft + mitral valve.

**Table 2:** List of candidate variables based on univariate logistic regression analysis compared with random forest.

Variable		Type	Unit	Univariate logistic regression analysis^a^			Random forest		
				OR (95% CI)	*P* value	Rank	Decrease of impurity, %	Nodes using that attribute, n	Rank
Age (years)		Continuous	Years	1.1 (1.03-1.07)	<.001	1^b^	43	3238	1
Mechanical ventilation >24 hours		Categorical	Yes/no	5.8 (3.9-8.6)	<.001	3	21	297	21
Preoperative creatinine clearance		Continuous	μmol/L	0.97 (0.96-0.98)	<.001	1^b^	39	2544	4
**Length of stay in the ICU^c^**		**Ordinal**	—^d^	—	—	**2**	**26**	**590**	**20**
	>72 hours	—	—	7.6 (4.9-11.9)	<.001	—	—	—	—
	24-72 hours	—	—	1.7 (0.9-2.8)	<.001	—	—	—	—
Procedure other than isolated CABG^e^		Categorical	Yes/no	2.9 (1.8-2.5)	<.001	6	28	370	15
Blood product within 48 hours		Categorical	Yes/no	2.9 (2.0-4.2)	<.001	5	28	452	14
Intraoperative TEE^f^		Categorical	Yes/No	2.0 (1.3-3.1)	.002	10	27	568	18
EURO II^g^ score		Continuous	Percent	1.07 (1.05-1.09)	<.001	17	41	2716	2
Preoperative hemoglobin		Continuous	gm/dL	0.98 (0.97-0.99)	<.001	1^b^	40	2766	3
Preoperative A-Fib^h^		Categorical	Yes/no	2.3 (1.4-3.6)	<.001	7	35	486	6
**Timing of IABP^i^**		**Ordinal**	—	—	—	**4**	**29**	**329**	**12**
	Preoperative	—	—	1.4 (0.6-2.9)	.42	—	—	—	—
	Intraoperative	—	—	6.8 (1.9-23.1)	.002	—	—	—	—
Intraoperative inotropes		Categorical	Yes/no	2.1 (1.4-3.0)	<.001	8	27	514	17
COPD^j^		Categorical	Yes/no	1.7 (1.1-2.7)	.02	14	33	689	9
CVD^k^		Categorical	Yes/no	1.8 (1.1-2.9)	.01	13	29	516	13
DM^l^		Categorical	Yes/no	0.9 (0.6-1.4)	.79	16	39	995	5
Frail		Categorical	Yes/no	2.0 (1.1-3.5)	.03	12	30	381	11
History of turn down		Categorical	Yes/no	8.2 (2.8-24.3)	<.001	1^b^	21	93	22
**EF^m^ categories**		**Ordinal**	—	—	—	**9**	**33**	**89**	**10**
	30%-50%	—	—	1.4 (0.9-2.1)	.18	—	—	—	—
	<30%	—	—	2.1 (1-4.2)	.04	—	—	—	—
Gender		Categorical	Yes/no	1.2 (0.8-1.9)	.47	16	35	752	7
**Aortic stenosis**		**Ordinal**	—	—	—	**14**	**26**	**899**	**16**
	Moderate	—	—	1.4 (0.6-2.8)	.43	—	—	—	—
	Severe	—	—	1.6 (1.1-2.5)	.01	—	—	—	
**Mitral insufficiency**		**Ordinal**	—	—	—	**15**	**26**	**899**	**19**
	Moderate	—	—	1.4 (0.9-2.1)	.07	—	—	—	—
	Severe	—	—	2.3 (1.02-4.6)	.03	—	—	—	—
Postoperative arrhythmias		Categorical	Yes/no	1.8 (1.3-2.8)	.002	11	34	746	8

^a^Analysis was done using univariate logistic regression with a *P* value of <.05 considered to be statistically significant.

^b^These variables were all equally ranked as 1st because they had almost equal odds ratios and a *P* value of <.001.

^c^ICU: intensive care unit.

^d^Not applicable.

^e^CABG: coronary artery bypass graft.

^f^TEE: transesophageal echo.

^g^EURO II: European System for Cardiac Operative Risk Evaluation II.

^h^A-Fib: atrial fibrillation.

^i^IABP: intra-aortic balloon pump.

^j^COPD: chronic obstructive pulmonary disease.

^k^CVD: cerebrovascular disease.

^l^DM: diabetes mellitus.

^m^EF: ejection fraction.

### Prediction Model’s Performance Evaluation

There exist several metrics to evaluate the performance of a predictive model, whereby predictive accuracy is the most commonly used metric as it relates a model’s ability to correctly identify observation assignments, irrespective of the class distribution. However, in the presence of a noted class imbalance in the dataset, this measure can be misleading because the minority class (positive cases in our dataset) has a smaller influence of the model’s output, and as such the model will tend to favor the majority class [47]. In our dataset, there is a significant imbalance of the outcome of interest distribution (delirium: 11.4% positive cases).

To provide a more robust evaluation of the prediction model’s performance, in the presence of the class imbalance in our dataset, we used the evaluation measures of F1 measure, ROC-AUC, and precision-recall curve area under the curve (PRC-AUC) [44,46,47,51,52]. The ROC-AUC was primarily used to assess the classifier’s general performance (model discrimination=how well the predicted risks distinguish between patients with and without disease) [64]. The F1 score was primarily used as the harmonic mean of precision and recall [46,52]. The F1 score provides the most reliable assessment of a model’s prediction performance, while considering the worst-case prediction scenario for a classifier (model calibration=evaluates the reliability of the estimated risks: if we predict 10%, on average 10/100 patients should have the disease) [64].

Sensitivity (recall) is considered a measure of completeness (the percentage of positive cases that have been correctly identified as positive). Positive predictive value (precision, PPV) is considered a measure of exactness (the percentage of cases labeled by the classifier as positive that are indeed positive) [46,52]. The PRC-AUC is a useful measure in the presence of class imbalance, and the outcome of interest is to identify the minority class [65,66]. The PRC identifies the PPV for each corresponding value on the sensitivity scale (model calibration). As the PRC is dependent on the class representation in the dataset, it provides a simple visual representation of the model’s performance across the whole spectrum of sensitivities. By doing so, it can aid in identifying the best model (based on the trade of being either exact vs complete, ideally optimizing both) [66]. In addition, the PRC enables comparing models at predetermined recall thresholds (eg, the best precision at 50% recall). This adds more fixability in choosing the best model based on the domain and problem in hand.

**Figure 1:**
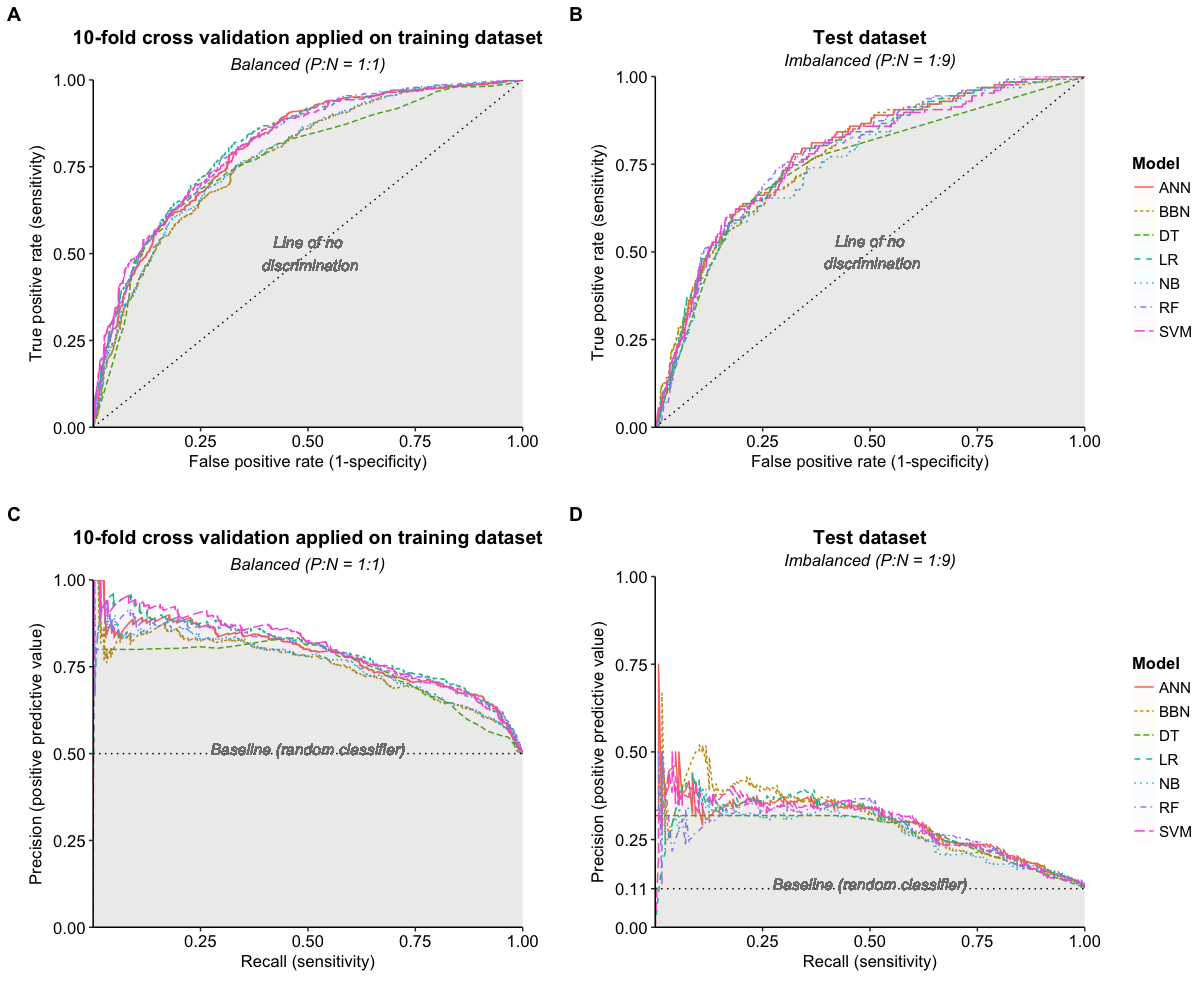
Receiver-operator curves (ROC) and precision-recall curves (PRC) for the training dataset using 10-fold cross-validation and test datasets. (A) ROC for training using 10-fold cross-validation. (B) ROC for test dataset. (C) PRC for training using 10-fold cross-validation. (D) PRC for test dataset. ANN: artificial neural networks; BBN: Bayesian belief networks; DT: J48 decision tree; LR: logistic regression; NB: naïve Bayesian; RF: random forest, SVM: support vector machines; P:N: positive to negative ratio.

As our primary interest was to identify patients who were more likely to develop delirium (minority class) while accounting for the class imbalance in the test dataset, we decided to evaluate the models using the ROC-AUC as a measure of the model discrimination in conjunction with F1 score and PRC-AUC as measures of the model calibration. Tables 3 and 4 present the prediction performance of all prediction models based on the test data. Figure 1 illustrates the ROC-AUCs and PRC-AUCs for the developed models.

When comparing the prediction performance using the ROC-AUC (Figure 1) for the test dataset, it may be noted that the prediction performance of all the prediction models on the test dataset is quite similar, except for DT, which was lower. This indicates that there is no obvious difference in the discriminative power of the classification models—that is, the ability of a model to distinguish between positive cases from negative ones. However, given the class imbalance in our dataset, this result might not be representative of a model’s true predictive power; hence, a further examination of the results was needed to identify the best performing model given the class imbalance.

As LR was the most commonly used algorithm to predict the manifestation of postoperative delirium in the medical literature [8,12,13,40,41,67-74], we developed a multivariate step-wise LR model that identified 8 variables as significant predictors of postoperative agitated delirium (Multimedia Appendix 2). The main purpose of developing the LR model was to give medical experts, who are not familiar with machine learning algorithms, an algorithm that they are acquainted with and use as a comparator.

In our study, for every 100 patients who developed delirium, the RF model had the best sensitivity and was able to correctly identify 72 patients (see Tables 3 and 4). The SVM model had the best PPV (out of 100 patients who were labeled positive by SVM, 30 were actually positive) and the best accuracy, specificity, and kappa. The PRC-AUC and F1 scores for SVM were the best out of all models (29.2% and 40.2%, respectively), with moderate discrimination (ROC-AUC=77.2 %). We also examined the relationship between precision (PPV) and recall (sensitivity) at different thresholds (see Table 5). At 50% sensitivity (recall), the RF model had the best precision, 37%). At 75% sensitivity (recall), RF was the best model with a precision of 25% followed by ANN with a PPV of 24%.

**Table 3:** Comparison of model’s performance metrics applied on the balanced training dataset using 10-fold cross-validation and the imbalanced test dataset to predict delirium after cardiac surgery. Performance metrics: accuracy, sensitivity, specificity, positive predictive value, negative predictive value, and Cohen kappa. All measures are reported out of 100% with standard deviation in brackets as a measure of variability.

Model		Accuracy	Δ^a^	Sensitivity	Δ	Specificity	Δ	PPV^b^	Δ	NPV^c^	Δ	Kappa	Δ
**Dataset: 10-fold cross-validation applied on the balanced training dataset (N=1014, delirium=50%)**													
	ANN^d^	71.7 (4.3)	ns^e^	71.8 (7)	+^f^	71.6 (7)	−^g^	71.7 (5)	−	71.7 (7)	−	43.3 (9)	ns
	BBN^h^	71.3 (4.4)	ns	72.2 (7)	+	71.2 (7)	−	69.9 (5)	−	71.3 (7)	−	43.1 (9)	ns
	DT^i^	70.1 (4.3)	ns	68.1 (7)	ns	72.9 (9)	ns	72.9 (5)	ns	72.6 (9)	ns	43.3 (8)	ns
	LR^j^	73.3 (4.4)	B^k^	69.8 (7)	B	76.7 (7)	B	75 (5)	B	75.6 (6)	B	44.5 (9)	B
	NB^l^	73.0 (4.2)	ns	64.8 (7)	ns	79.5 (5)	+	74.4 (5)	ns	79.5 (5)	+	42.9 (8)	ns
	RF^m^	72.5 (4.4)	ns	74.3 (7)	+	71.7 (7)	−	72.1 (4)	ns	72.8 (7)	−	45.7 (9)	ns
	SVM^n^	71.3 (4.5)	ns	60.2 (8)	−	83.8 (5)	+	77.8 (5)	+	83.1 (5)	+	43.2 (9)	ns
**Dataset: Imbalanced test dataset (N=1117, delirium=11.4%)**													
	ANN	74.3 (3.2)	ns	67.7 (5)	+	72.9 (5)	ns	24.3 (14)	ns	94.6 (5)	ns	22.85 (9)	ns
	BBN	74.1 (3.8)	ns	68.7 (9)	+	70.8 (9)	−	22.9 (15)	ns	94.5 (6)	ns	21.81 (11)	ns
	DT	74.4 (5.4)	ns	66.9 (10)	+	75.4 (10)	ns	25.8 (17)	ns	94.7 (10)	ns	24.97 (13)	ns
	LR	75.6 (4.7)	B	64.6 (9)	B	77.1 (7)	B	26.5 (16)	B	94.4 (8)	B	22.6 (13)	B
	NB	71.7 (3.1)	−	66.1 (12)	ns	72.4 (8)	−	23.5 (18)	ns	94.3 (9)	ns	21.55 (10)	ns
	RF	75.4 (3.4)	ns	72.4 (4)	+	72.4 (4)	−	25.2 (8)	+	95.3 (4)	+	24.69 (7)	ns
	SVM	78.9 (2.1)	+	62.2 (4)	ns	81.1(3.2)	**+**	29.7 (12)	+	94.4 (6)	ns	29.33 (9)	+

^a^Change compared to base model (B).

^b^PPV: positive predictive value.

^c^NPV: negative predictive value.

^d^ANN: artificial neural networks.

^e^ns: not a statistically significant change in performance (*P*≥.05).

^f^Statistically significant improvement of performance metric (*P*<.05).

^g^Statistically significant deterioration of performance metric (*P*<.05).

^h^BBN: Bayesian belief networks.

^i^DT: J48 decision tree.

^j^LR: logistic regression.

^k^B: base comparator (reference) algorithm.

^l^NB: naïve Bayesian.

^m^RF: random forest.

^n^SVM: support vector machines.

**Table 4:** Comparison of model’s performance metrics applied on the balanced training dataset using 10-fold cross-validation and the imbalanced test dataset to predict delirium after cardiac surgery. Performance metrics: receiver operator curve-area under the curve, harmonic mean of precision and recall, and precision-recall curve-area under the curve. All measures are reported out of 100% with standard deviation in brackets as a measure of variability.

Model		ROC-AUC^a^			F1 score^b^													PRC-AUC^c^										
					Yes^d^		Δ^e^		No^f^		Δ		Avg^g^		Δ		Yes		Δ		No		Δ		Avg		Δ	
**Dataset: 10-fold cross-validation applied on the balanced training dataset (N=1014, delirium=50%)**																												
	ANN^h^		80.4 (4)	ns^i^		71.7 (5)		ns		71.7 (5)		ns		71.7 (5)		ns		78.5 (5)		ns		80.1 (5)		ns		79.3 (5)		ns
	BBN^j^		77.4 (4)	−^k^		70.1 (5)		ns		69.1 (5)		ns		69.6 (5)		ns		75.3 (5)		ns		77.3 (5)		ns		76.3 (5)		−
	DT^l^		77.2 (4)	ns		70.9 (4)		ns		72.4 (4)		ns		71.7 (4)		ns		74.4 (5)		ns		73.8 (5)		ns		73.8 (5)		−
	LR^m^		81.4 (4)	B^n^		72.3 (5)		B		74.2 (5)		B		73.2 (5)		B		79.8 (5)		B		81 (5)		B		80.4 (5)		B
	NB^o^		79.9 (4)	ns		72.7 (5)		ns		73.2 (5)		ns		73 (5)		ns		78.1 (5)		ns		79.8 (5)		ns		78.9 (5)		ns
	RF^p^		81.3 (4)	ns		74.1 (5)		ns		72.6 (5)		ns		73.3 (5)		ns		78.8 (5)		ns		81 (5)		ns		79.9 (5)		ns
	SVM^q^		81.1 (5)	ns		67.2 (6)		−		74.4 (6)		ns		71.1 (6)		−		80.4 (5)		ns		80.5 (5)		ns		80.4 (5)		ns
**Dataset: Imbalanced test dataset (N=1117, delirium=11.4%)**																												
	ANN		78.2 (6)	ns		35.8 (9)		ns		82.4 (9)		ns		77.1 (9)		ns		30.4 (9)		+^r^		96.2 (9)		ns		88.7 (9)		ns
	BBN		77.3 (6)	ns		34.3 (8)		ns		82.9 (8)		ns		76.6 (8)		ns		30.7 (8)		+		95.8 (8)		ns		88.4 (8)		ns
	DT		74.6 (7)	−		37.3 (8)		ns		83.9 (8)		ns		78.6 (8)		ns		25.3 (8)		ns		94.3 (8)		ns		86.5 (8)		ns
	LR		77.5 (5)	B		37.6 (11)		B		84.9 (11)		B		79.5 (11)		B		27.1 (10)		B		97.1 (10)		B		88.4 (10)		B
	NB		75.6 (8)	ns		34.7 (10)		ns		81.9 (10)		ns		76.6 (10)		ns		28.7 (9)		ns		95.6 (9)		ns		88.0 (9)		ns
	RF		78.0 (4)	ns		37.4 (8)		ns		82.3 (8)		ns		77.2 (8)		ns		28.3 (8)		ns		96.3 (8)		ns		88.6 (8)		ns
	SVM		77.2 (6)	ns		40.2 (7)		+		87.2 (7)		+		81.9 (7)		+		29.6 (9)		+		96.0 (9)		ns		88.4 (9)		ns

^a^ROC-AUC: receiver operator curve-area under the curve.

^b^F1 score: harmonic mean of precision and recall.

^c^PRC-AUC: precision-recall curve-area under the curve.

^d^Yes: positive instances or patients who developed delirium.

^e^Change compared to base model (B)

^f^No: negative instances or patients who did not develop delirium.

^g^Avg: weighted average measured as the sum of all values in that metric, each weighted according to the number of instances with that particular class label by multiplying that value by the number of instances in that class, then divided by the total number of instances in the dataset.

^h^ANN: artificial neural networks.

^i^ns: not a statistically significant change in performance (*P*≥.05).

^j^BBN: Bayesian belief networks.

^k^Statistically significant deterioration of performance metric (*P*<.05).

^l^DT: J48 decision tree.

^m^LR: logistic regression.

^n^B: base comparator (reference) algorithm.

^o^NB: naïve Bayesian.

^p^RF: random forest.

^q^SVM: support vector machines.

^r^Statistically significant improvement of performance metric (*P*<.05).

**Table 5:** Precision of each model for all datasets at different recall thresholds.

Recall threshold (%)		Model precision (%)						
		ANN^a^	BBN^b^	DT^c^	LR^d^	NB^e^	RF^f^	SVM^g^
**Dataset: Training with 10-fold cross-validation**								
	~25	87	83	81	88	85	87	91
	~50	80	78	81	82	78	83	83
	~75	71	70	69	73	70	72	72
**Dataset: Test**								
	~25	35	40	31	36	32	32	36
	~50	34	33	30	34	31	37	34
	~75	24	22	21	23	20	25	23

^a^ANN: artificial neural networks.

^b^BBN: Bayesian belief networks.

^c^DT: J48 decision tree.

^d^LR: logistic regression.

^e^NB: naïve Bayesian.

^f^RF: random forest.

^g^SVM: support vector machine.

On the basis of our experiments using the PRC-AUC and PRC analysis, the RF and ANN models demonstrated the ability to distinguish patients at risk of developing delirium (minority class) when compared with the other models. ANN is considered to be a *black box* as it is difficult to explain, especially to people who are nonexperts, not familiar with the principles and motivation behind the ANN algorithm, and do not know how the algorithm reaches its decision and activation thresholds. However, major work has been conducted over the last decade and is still ongoing to enhance the expandability of ANN by unlocking the black box to allow accountability [75-77]. Numerous techniques have been developed and were successfully applied [78-80], giving some transparency to the model and making it more human interpretable.

## Discussion

### Principal Findings

Patients undergo high-risk interventions with the expectation of improving their quality of life. It is highly undesirable that any medical intervention, inadvertently, negatively impacts their cognitive functions and in turn quality of life, especially if an adverse outcome is preventable.

With the paradigm shift in health care emphasizing the patient’s quality of life after an intervention [81], innovative approaches are needed to both pre-emptively identify and effectively treat delirium. Given the availability of long-term surgical outcome data and advance machine learning methods, it is now possible to investigate the formulation of data-driven prediction models to pre-emptively identify patients susceptible to postsurgery delirium. LR-based prediction models to detect delirium have been developed using patient data from electronic medical records—in one study advanced text mining has been applied to abstract relevant data from clinical notes [82], and in another study attribute-based triggers were used [57]. We contend that with the availability of large volumes of patient data (before, during, and after the medical intervention), there are practical opportunities to develop data-driven prediction models to detect postoperative delirium in patients. Such artificial intelligence–based machine learning–based models are quite capable of identifying hidden yet important relations among variables and representing them in terms of a mathematical model that can be applied to classify/predict the output for new scenario. The artificial intelligence-based machine learning approach is rather different from the traditional statistical data analysis approaches; however, recently such methods have been applied to improve early and precise detection of diseases [16,21,25,27-29], including the prediction of outcomes after cardiac surgery [22,32,83].

In our study, we investigated the development of delirium prediction models using long-term (over 5 years) surgical outcomes data for over 5000 patients. We developed several prediction models, while addressing the underlying class imbalance issue, and compared their performance on an independent test set. Except for SVM (ROC-AUC=71.7%), the ROC-AUC of the predictive models was at least 75%, indicating a good general performance by predicting the correct classification most of the time [84,85]. Using the F1 score and the PRC-AUC, which are more sensitive to class imbalance, we were able to demonstrate that the SVM followed by the BBN models offered the best prediction performance in correctly identifying adult patients at risk of developing agitated delirium after cardiac surgery (F1 score: 40.2 and 34.4 and PRC-AUC: 30.7 and 29.6; respectively).

Our predictive models had a worse performance when compared with the findings of Kumar et al [39] (ROC-AUC of the RF model ~91%). Although they argue that their data were imbalanced, they used the ROC-AUC as their evaluation metric, which does not consider the class imbalance. On the other hand, PRC-AUC inherently accounts for class distribution (the probability is conditioned on the model estimate of the class label, which will vary if the model is applied on a population with different baseline distributions). It is more useful if the goal is improving the prediction of *positive* class in an imbalanced population with known baseline probability (eg, document retrieval, fraud detection, and medical complications) [44,46,48,51,66].

Compared with the findings of Corradi et al [42] (ROC-AUC of the RF model ~91% and PRC-AUC ~61 %), our model was worse. Although they included a lot of physiological parameters, they did not include any laboratory parameters. In addition, they applied the algorithm on all patients within the study period (medical and surgical). Most of the variables used were correlated—that is, they were a function of each other (eg, RASS and mechanical ventilation, RASS score and vasopressors, and dementia and the Charleston Comorbidity Index)—which likely impacted the generalizability of the model.

The paper published by Davoudi et al [40] is the only paper that is closely related to our work as they were specifically addressing the question of predicting delirium after major surgery and had a large cohort of patients who underwent cardiothoracic surgery (6890 patients, 13%). They were able to achieve an ROC-AUC ranging from 79% to 86%, which was close to the ROC-AUC we were able to achieve (71.7%-78%). Unfortunately, it is not clear what type of delirium they were capturing and the urgency of surgery these patients were undergoing. Also, only 13% of these patients underwent cardiothoracic surgery. They mainly relied on the ROC-AUC to compare the model’s performance, which is insensitive to the target class imbalance.

Lee et al [41] conducted a unique systematic review in 2017, addressing the issue of predictive models for discovering delirium after cardiac surgery. They were only able to identify 3 high-quality models (Katznelson, Original PRE-DELIRIC, and the recalibrated PRE-DELIRIC). As the original PRE-DELIRIC was recently externally validated, they externally validated the Katznelson and recalibrated PRE-DELIRIC model on a local population dataset of 600 patients. Several metrics were used to evaluate the model’s discrimination and calibration. All metrics for recalibrated PRE-DELIRIC model outperformed the Katznelson model (see Multimedia Appendix 1). However, these metrics cannot distinguish clinical utility. To identify clinical utility of these models, they performed DCA to ascertain the clinical utility of each model. The main advantage of DCA is that it incorporates preferences (patient or physician) represented as threshold probability of choosing or denying a treatment, across a range of probabilities [41]. The net benefit (the expected benefit of offering or denying a treatment at that threshold) of each algorithm was evaluated. Based on the DCA analysis, both models had limited clinical utility, with the recalibrated PRE-DELIRIC having marginally better performance at low thresholds between 20% and 40%. Regrettably, they used already validated models that are based on LR. They mentioned very limited information about the validation cohort (such as mean age, gender distribution, and type of cardiac surgery). In addition, they did not address the significant class imbalance (delirium=13.8%). Finally, the use of DCA to evaluate clinical utility of the models is very innovative but it can be only applied to evaluate models that were developed by the same algorithm but have different parameters. Its applicability across different modeling algorithms is still not clear. One of the essential assumptions of DCA is that the predicted probability and threshold probability are independent. In the case of delirium, it would be very difficult to assert that independence, as delirium is multifactorial, and there is no clear mechanism to its development. Violating this assumption might significantly affect the results and interpretation of the DCA.

To our knowledge, this is the first paper that explicitly attempts to develop several predictive models using machine learning methodology and compare their performance for the sole purpose of proactively predicting agitated delirium in adult patients undergoing cardiac surgery. A notable aspect of our work is the use of multiple performance evaluation measures to evaluate the different facets of a prediction model with respect to its prediction performance. We demonstrated the importance of using different metrics when analyzing model’s performance (eg, F1 score and PRC-AUC) and the importance of visual analysis of the curves across different probabilities (eg, PRC). Using a static or single measure, like ROC-AUC or accuracy, might lead to false assumptions and incorrect decisions, especially in the presence of class imbalance in the dataset [66].

An important factor in the selection of a prediction model is its interpretability (clarity) to the users (especially health care providers) who are particularly keen to know the basis for a recommendation/decision when it is derived from a computational model. One of the drawbacks of ANN and SVM is that they are not easy to explain, that is, how the output was produced (ie, they are regarded as black box models). This inability to explain the model and its predictions tends to raise a degree of skepticism among health care practitioners regarding the prediction produced [46,52,56]. However, the application of additional methods to decipher the ANN and SVM models’ decision logic in terms of understandable production rules that illustrate a correlation between clinical attribute values and the output class can increase their acceptance and subsequent use by medical practitioners [75-80]. Other machine learning methods, such as the BBN model provides a simple but elegant graphical representation of the problem space that can be interpreted by health care professionals.

Predicting delirium is a challenging problem, but with a significant health outcome and system use impact. Given the complexity of how and why delirium manifests in certain patients, the ability to correctly identify if not all but even a fair number of the potential patients who are at risk of developing delirium will be a significant improvement from the current state where patients are diagnosed with delirium only after it starts, and hence, the administration of appropriate interventions is delayed. To address this challenging problem, we investigated the application of machine learning methods to predict postoperative delirium after cardiac surgery. Our methodology involved addressing the target class imbalance and employing appropriate evaluation metrics to measure the prediction performance from a clinical utility perspective. We argue that with the increased use of eHealth records and auxiliary data collection tools, the volume of health data being collected is reaching the level of *big data*. This brings relief to the need to apply advance machine learning techniques to analyze the data for improved and effective data-driven decision support [86,87] that would enable timely intervention for negative outcomes [5,56,87] to improve health outcomes and in turn enhance patient safety and satisfaction.

### Limitations

We recognize that our study has certain limitations. First, as postoperative complications (including delirium) in our database are captured as binary outcomes (yes/no) but without a time stamp, it was hard to determine if agitated delirium was a secondary phenomenon (eg, because of infection, uncontrolled pain, and prolonged mechanical ventilation) or because of a pre-existing medical comorbidity. Second, the prevalence of agitated delirium was only 11.4%. This low representation is most likely because of the definition of delirium in the source database (only agitated subtype). This can potentially limit the ability to generalize the developed models to other types of delirium [10,11]. Third, there exist more advance machine learning software than what were available in WEKA, but we chose WEKA because of its open source, flexibility, and ease of use [54]; and finally, the study is based on a retrospective design and hence may suffer from the pitfalls associated with such a design.

### Clinical Equipoise and Key Messages

The key messages of this paper are as follows:

- From a clinical standpoint:
  - Patients undergoing cardiovascular surgical procedures are at higher risk of developing agitated delirium due to several factors, including surgical complexity, comorbidities, and age [7,8].
  - Preventing delirium should be the goal, especially if patients at risk were identified. This will mitigate its negative sequalae and improve the patient’s quality of life. Some of the proposed preventive interventions that have been shown to reduce the incidence of delirium in high-risk patients include early mobilization, use of patient’s personal aids (reading glasses, hearing aid, etc), pharmacological interventions (the use of less sedatives and addressing pain), and improving sleep environment especially in the intensive care [38,88-91].
- From a predictive modeling perspective:
  - Addressing class imbalance on the training dataset (a common feature of medical datasets) could enhance the machine learning model’s performance in identifying patients likely to develop postoperative delirium.
  - Keeping an open mind and exploring different modeling methodologies will enable the selection of the most appropriate model that can generate the best results.
  - The PRC offers a more intuitive and direct measure of the model performance that is representative of its true performance, especially in the presence of class imbalance.

### Conclusions and Future Research

Postoperative agitated delirium is associated with major morbidity that impacts the patient postoperative recovery. Cardiac surgery patients are at high risk of developing postoperative delirium. To improve health outcomes of cardiac surgery, the current approach to address the effects of delirium is a preventive program of care [88-91], such as ABCDE, which involves awakening and breathing coordination for liberation from sedation and mechanical ventilation, choosing sedatives that are less likely to increase risk of delirium, delirium management, and finally, early mobility and exercise [36]. As much as the ABCDE approach provides a road map of how to manage delirium, it does not provide mechanisms to identify patients at risk of developing delirium. Hence, the ABCDE approach serves as an after-the-event management strategy, while leaving a gap in terms of a proactive prevention strategy for delirium. Our ability to predict delirium in patients, and in turn proactively administer therapeutic and behavioral therapies to mitigate the negative effects of delirium, will lead to significant improvements in health outcomes, patient satisfaction and quality of life, and health system cost saving.

In this study, we pursued the development of prediction models using preoperative clinical data to establish a mapping between the patient’s preoperative clinical variables and the onset of postoperative delirium. We investigated machine learning methods to develop a viable postoperative delirium prediction model which can be operationalized in a clinical setting as a delirium screening tool to proactively identify patients at risk of developing postcardiac surgery agitated delirium. We posit that the use and operationalization of delirium predictive model can significantly reduce the incidence of delirium by enabling the administration of preventive measures in a timely manner. In this paper, we presented work detailing the development of data-driven delirium prediction models with a reasonable accuracy. Furthermore, the work contributes 3 findings that are useful for future efforts to develop advanced delirium prediction models—that is, (1) addressing class imbalance on the training dataset will enhance the machine learning model’s performance in identifying patients likely to develop postoperative delirium, (2) when evaluating the model’s performance, selecting unsuitable measures can influence model interpretation and its utility, and (3) the PRC offers a more intuitive and direct measure of the model’s performance that is representative of its true performance, especially in the presence of class imbalance.

In our future research, we will attempt to apply feature extraction to identify key features to enhance the model’s performance. At the same time, we will attempt to isolate modifiable features that are clinically relevant so that personalized interventions can be started in a timely fashion. We will also attempt to apply evolutionary computations to optimize classifiers parameters. Another interesting application is the use of deep learning methods to create new features or feature sets to boost the model’s performance and accuracy.

In conclusion, we argue that any improvement in our ability to predict delirium using prediction models, even if numerically small, is of consequential clinical significance—this situation is like 2 drugs that have the same treatment profile, but one drug has fewer side effects, and hence, the ability to precisely select the right drug has an impact on patient safety. When dealing with complex medical problems, such as delirium, we posit that the application of advanced machine learning methods might actually improve disease prediction capabilities which in turn will enhance opportunities for preventive, personalized, and precise medical interventions that would improve the patient’s quality of life after surgery.

## Appendix

Multimedia Appendix 1 Summary of relevant related work.

Multimedia Appendix 2 Machine learning methodology and forest plot for logistic regression.

Multimedia Appendix 3 Setting of the prediction models and the optimization steps that were applied on the used algorithms.

## Abbreviations

ANN: artificial neural networks
BBN: Bayesian belief networks
CABG: coronary artery bypass graft
CAM: confusion assessment method
CAM-ICU: confusion assessment method for the intensive care unit
DCA: decision curve analysis
DT: decision trees
eHealth: electronic health
ICU: intensive care unit
LR: logistic regression
MHC: Maritime Heart Center
NB: naïve Bayesian
OOB: out-of-bag
OR: odds ratio
PPV: positive predictive value (precision)
PRC-AUC: precision-recall curve-area under the curve
QEII HSC: Queen Elizabeth II Health Sciences Center
RASS: Richmond Agitation-Sedation Scale
RF: random forest
ROC-AUC: receiver operator curve-area under the curve
RUS: random majority undersampling
SVM: support vector machines
WEKA: Waikato Environment for Knowledge Acquisition
