# Supplementary material for: Exploiting Machine Learning Algorithms and Methods for the Prediction of Agitated Delirium After Cardiac Surgery: Models Development and Validation Study
Source: JMIR Med Inform. 2019 Oct 23;7(4):e14993. doi: 10.2196/14993 (PMC6913743; doi:10.2196/14993)
Supplement: Multimedia Appendix 1 [file medinform_v7i4e14993_app1.docx]

APPENDIX 1:

Table A1.1: Summary of relevant related work

| Year | Author | Population | Specific to Cardiac Surgery | Patients with Delirium % |
| --- | --- | --- | --- | --- |
| 1993 | Tinetti et al. [1] | - Hospitalized general medical patients 70 years or older who did not have dementia or delirium at admission. | No | 25% |
| 1996 | Inouye & Charpentier [2] | - General medical wards, university teaching hospital. | No | 18% |
| 1996 | O’Keeffe & Lavan [3] | - Patients admitted to a Geriatrics Unit (Mean Age 80 years) | No | 25-28% |
| 2007 | Inouye et al. [4] | - Patients 70 years or older admitted to the hospital without delirium and surviving to discharge. - From the general medical units of an academic teaching hospital | No | 11.8% |
| 2009 | Katznelson et al. [7] | - Patients undergoing Cardiac Surgery - Admitted to a mixed ICU | Yes | 11.5% |
| 2010 | Afonso et al. [9] | - Patients undergoing Cardiac Surgery - Admitted to a cardiac surgical ICU | Yes | 34% |
| 2012 | Isfandiaty et al. [11] | - Elderly patients (aged 60 years and older) who were hospitalized in Internal Medicine Ward and Acute Geriatric Ward | No | 18.8% |
| 2012 | Bakker et al. [17] | - Patients of 70 years and older who underwent cardiac surgery | Yes | 31% |
| 2012 | van den Boogaard [21] | - 3056 intensive care patients aged 18 years or over. - Mixed ICU | No | 30% |
| 2014 | Carrasco et al. [30] | - Medical Inpatients 65 years and older | No | 31% |
| 2017 | Kramer et al. [33] | - Hospitalized patients from geronto-psychiatry and internal medicine department | No | 26% |
| 2017 | Kumar et al. [45] | - Patients age 18-80 undergoing Cardiac surgery in a single center | Yes | 17.5% |
| 2017 | Davoudi, et al. [46] | - All patients 18 years of age and older who were admitted for - longer than 24 hours following any type of inpatient operative - procedure | No | 3.1 |
| 2017 | Lee et al. [50] | - Externally validate and assess the performance of all published validated risk prediction models of delirium, using the CAM-ICU assessment tool for detection of delirium. - Two models were assessed: Katznelson prediction model and the recalibrated PRE-DELIRIC prediction model - Validation using 600 adult patients undergoing cardiac surgery | Yes | 13.8% |
| 2018 | Corradi et al. [51] | - All adult patients admitted to a single institution | No | 6% |
| 2019 | Chaiwat et al. [52] | - adults aged over 18 years who had undergone an operation within the preceding week and who had been admitted to a Surgical ICU for a period that was expected to be longer than 24 hours | No | 24.4% |
| *ENET= Elastic Net, KNN= K−Nearest Neighbor, LDA= Linear Discriminant Analysis, LR= Logistic Regression, NN= Neural Network, PRC-AUC= Area Under the Precision Recall Curve, GAM: Generalized Additive Model, NB: Naïve Bayes, ROC-AUC= Area Under the Receiver Operator Curve, RF= Random Forests, SMOTE: Synthetic Minority Oversampling Technique, SVM= Support Vector Machine, XGB: Extreme Gradient Boosting,* | | | | |

Table A1.2: Summary of relevant related work

| Year | Author | Training Ste Size | Test Set Size | Algorithm’s Used | Addressed Class Imbalance | Model Assessment | Best Results |
| --- | --- | --- | --- | --- | --- | --- | --- |
| 1993 | Tinetti et al. [1] | 107 | 174 | LR | No | - ROC-AUC | - ROC-AUC- on Test Set= 74% |
| 1996 | Inouye & Charpentier [2] | 196 | 312 | LR | No | - Goodness of fit | - Not reported |
| 1996 | O’Keeffe & Lavan [3] | 100 | 84 | LR | No | - ROC-AUC | - ROC-AUC- on Test Set = 75% |
| 2007 | Inouye et al. [4] | 491 | 461 | LR | No | - ROC-AUC | - ROC-AUC- on Test Set = 75% |
| 2009 | Katznelson et al. [7] | 1059 | Bootstrap | LR | No | - ROC-AUC | - ROC-AUC- on Bootstrap Training Set = 77% |
| 2010 | Afonso et al. [9] | 112 | Not used | LR | No | - Not reported | - NA |
| 2012 | Isfandiaty et al. [11] | 457 | Not used | LR | No | - Hosmer-Lemeshow test - ROC-AUC | - ROC-AUC – on Training Set= 82% |
| 2012 | Bakker et al. [17] | 201 | Not used | LR | No | - ROC-AUC | - ROC-AUC – on Training Set= 71% |
| 2012 | van den Boogaard [21] | 1613 | 894 | LR | No | - ROC-AUC | - ROC-AUC - on Test Set = 84% |
| 2014 | Carrasco et al. [30] | 374 | 104 | LR | No | - ROC-AUC | - ROC-AUC - on Test Set = 78% |
| 2017 | Kramer et al. [33] | 6420 | 2141 | RF, LDA, ENET, NN, KNN, SVM | No | - Sensitivity - Specificity - Accuracy - Cohen’s Kappa - ROC-AUC | - ROC-AUC for the RF model - on Test Set = 91% |
| 2017 | Kumar et al. [45] | 120 | Not used | LR | No | - Not reported | - NA |
| 2017 | Davoudi, et al. [46] | 51457 | 10291 | LR, GAM, RF, SVM, NN, XGB, NB | Yes  (used data level manipulation: SMOTE and Under sampling) | - ROC-AUC | - ROC-AUC- on normal Test Set: GAM= 86% % - ROC-AUC- on Test Set with SMOTE: LR & RF = 86% - ROC-AUC- on Test Set with Under sampling RF = 85% |
| 2017 | Lee et al. [50] | NA | 600 | LR | No | - ROC-AUC - Hosmer–Lemeshow (HL) test - Nagelkerke’s R^2^ - Brier score - Decision Curve analysis (DCA) | - Katznelson prediction model: ROC-AUC= 62%,   HL test= 0.04, R2= 0.037, Brier score= 0.117   - Recalibrated PRE-DELIRIC prediction model: ROC-AUC= 75%,   HL test= 0.99, R^2^= 0.19, Brier score= 0.104   - Using DCA, the clinical usefulness of both models   appears clinically limited |
| 2018 | Corradi et al. [51] | 51240 | 12798 | RF | No | - ROC-AUC - PRC-AUC - Accuracy - F-Score | - ROC-AUC - on Test Set = 91% - PRC-AUC - on Test Set = 60% |
| 2019 | Chaiwat et al. [52] | 250 | Bootstrap | LR | No | - ROC-AUC | - ROC-AUC - on Bootstrap Training Set = 82% |
| *ENET= Elastic Net, KNN= K−Nearest Neighbor, LDA= Linear Discriminant Analysis, LR= Logistic Regression, NN= Neural Network, PRC-AUC= Area Under the Precision Recall Curve, GAM: Generalized Additive Model, NB: Naïve Bayes, ROC-AUC= Area Under the Receiver Operator Curve, RF= Random Forests, SMOTE: Synthetic Minority Oversampling Technique, SVM= Support Vector Machine, XGB: Extreme Gradient Boosting,* | | | | | | | |
