# Supplementary material for: Exploiting Machine Learning Algorithms and Methods for the Prediction of Agitated Delirium After Cardiac Surgery: Models Development and Validation Study
Source: JMIR Med Inform. 2019 Oct 23;7(4):e14993. doi: 10.2196/14993 (PMC6913743; doi:10.2196/14993)
Supplement: Multimedia Appendix 2 [file medinform_v7i4e14993_app2.docx]

APPENDIX 2:

**
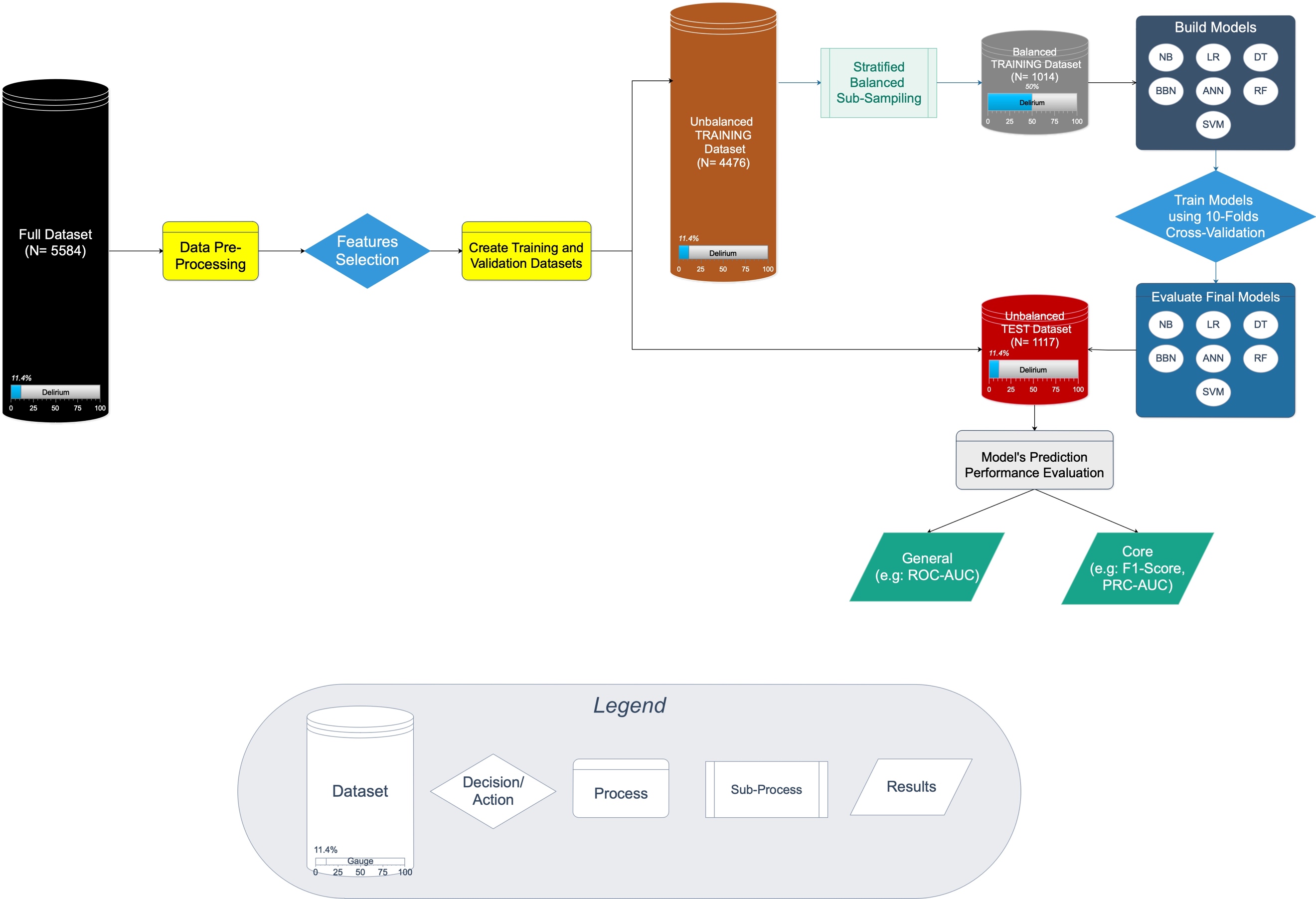
**

Figure 2.1: Data mining methodology adapted in our project

*ANN: Artificial Neural Networks, BBN: Bayesian Belief Networks, DT: J48 Decision Trees, F1-Score: Harmonic Mean of Precision and Recall, LR: Logistic Regression, NB: Naïve Bayesian, PRC-AUC: Precision-Recall Curve Area Under the Curve, RF: Random Forrest, ROC-AUC: Receiver Operating Characteristic Area Under the Curve, SVM: Support Vector Machines.*


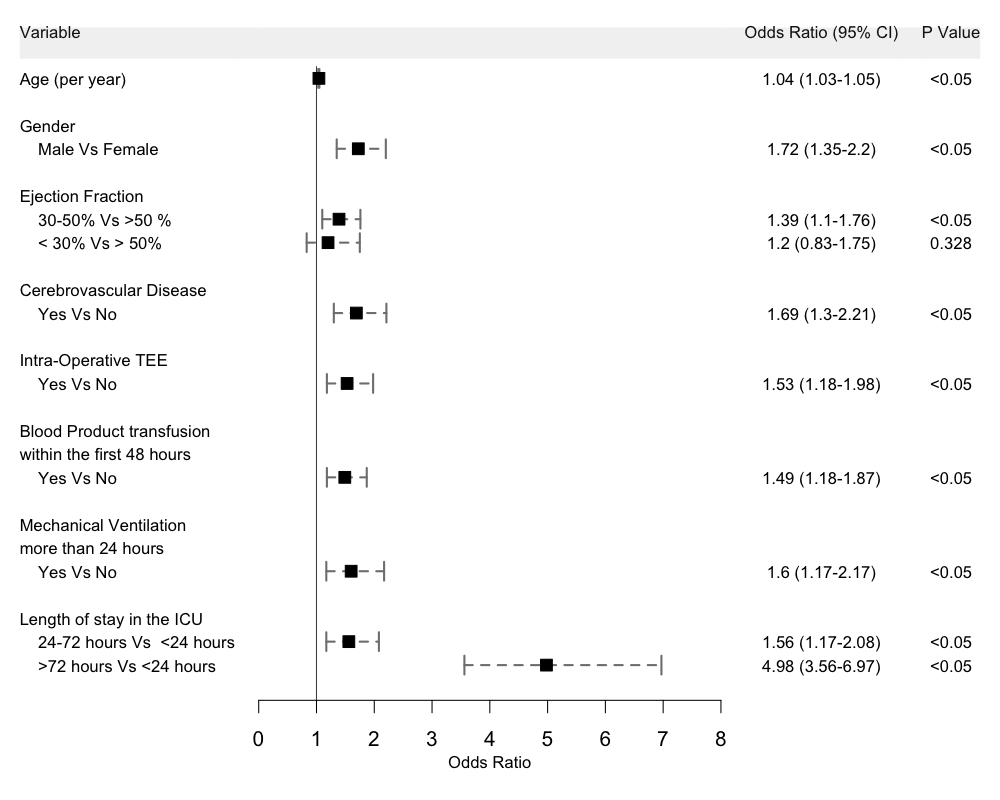


Figure 2.2: Forest Plot for the Multivariate Stepwise Logistic Regression Model Odds Ratio for Predicting Agitated Delirium after Cardiac Surgery in Adult.

*ICU: Intensive Care Unit, TEE: Trans-esophageal Echocardiography, CI: Confidence Interval*
