# Supplementary material for: Exploiting Machine Learning Algorithms and Methods for the Prediction of Agitated Delirium After Cardiac Surgery: Models Development and Validation Study
Source: JMIR Med Inform. 2019 Oct 23;7(4):e14993. doi: 10.2196/14993 (PMC6913743; doi:10.2196/14993)
Supplement: Multimedia Appendix 3 [file medinform_v7i4e14993_app3.docx]

APPENDIX 3:

Prediction Model Parameters Setting

### ANN Prediction Model:

In terms of ANN, we used the supervised learning Back Propagation (BP) algorithm to develop a prediction model. The optimal BP ANN configuration was determined through a series of experiments with different network configurations (i.e. hidden layer size, learning rate, momentum etc.). The range of values for the BP ANN parameters were investigated as follows: Hidden layer unites from 3-24 units, learning rate from 0.1-0.3, momentum from 0-0.2 and decay 0-1. All ANN were trained on the same dataset—i.e. 21 patient attributes and a binary output of Delirium Yes/No. Based on the prediction performance and the ability to generalize new data, the optimal ANN configuration was 21 input units, 3 hidden units, 1 output unit, sigmoid learning function, learning rate of 0.275, momentum of 0 and delay set to false[1-4].

### Naïve Bayesian & Belief Network Models:

For NB, the default setting was used. BBN can identify hidden relationships and dependencies between variables based on the structure of the data, without any prior knowledge or influence from the analyst. In this regard, we used the (Look Ahead in a Good Direction) Hill Climbing algorithm[2, 5] that uses a stochastic approach to develop a classification model (as a belief network) by disregarding the order of the attributes, looking ahead a specified number of steps, examining several network structures and then choosing the best one to represent the dataset. Setting the number of parents to a node in a BBN is a hard task, for which no formal method exists, rather we used a combination of heuristics, experiments and domain knowledge to arrive at the most optimal BNN model configuration, where number of parents = 2. Alpha was set to 0.1 to simulate maximum likelihood estimate[1-4, 6-10].

### Decision Trees and Random Forrest Models:

For DT, the J48 consolidated algorithm was used. We adjusted the minimum number of instances in each leaf to 25 to avoid overfitting. With regards to the RF algorithm, we limited the maximum depth of a tree to 10 to avoid overfitting[1-4].

### Logistic Regression Predictive Model:

To develop the logistic regression model all significant, independent risk factors for post-operative agitated delirium were used. A step wise logistic regression model was used with a *p-value*=0.05 and 95% CI. The multivariate logistic regression model only identified 8 attributes as significant predictors of post-operative agitated delirium (**Figure 2**). Odds ratio (OR) were extracted and used to measure the association and impact of each attribute on delirium[1-4, 11, 12].

### Support Vector Machine Model:

To optimize the results of the SVM model, we started by converting all categorical attributes into numeric representation. Then we scaled all numerical attributes between (-1 to +1). We chose the radial base kernel as it is capable of handling nonlinear relationships, using a grid search and 10-fold cross validation, where the optimal C= 1 and γ = 0.01 of the C-SVM in WEKA[3, 4, 13, 14].

REFERENCES

[1] Han J, Kamber M, Pei J. Data Mining: Concepts and Techniques: Morgan Kaufmann Publishers Inc.; 2011.

[2] Ian H. Witten FE, Mark A. Hall. Data Mining: Practical Machine Learning Tools and Techniques. Third Edition ed: Morgan Kaufmann Publishers; 2011.

[3] Hastie TT, Robert; Friedman, Jerome. The Elements of Statistical Learning Data Mining, Inference, and Prediction: Springer; 2008.

[4] Hall M, Frank E, Holmes G, Pfahringer B, Reutemann P, Witten IH. The WEKA data mining software. SIGKDD Explor Newsl. 3.7.10 ed2009. p. 10-8.

[5] BOUCKAERT R. Bayesian Network Classifiers in Weka for Version 3-5-7, University of Waikato. <http://www> cs waikato ac nz/~ remco/weka bn pdf. 2008.

[6] Albert J. Bayesian Computation with R: Springer; 2009.

[7] Bouckaert RR. Bayesian network classifiers in weka: Department of Computer Science, University of Waikato; 2004.

[8] Bernardo JM, Smith AF. Bayesian theory: John Wiley & Sons; 2009.

[9] Koivisto M, Sood K. Exact Bayesian structure discovery in Bayesian networks. The Journal of Machine Learning Research. 2004;5:549-73.

[10] Koch K-R. Introduction to Bayesian Statistics. 2 ed: Springer; 2007.

[11] Menard S. Applied logistic regression analysis: Sage; 2002.

[12] Stoltzfus JC. Logistic regression: a brief primer. Academic Emergency Medicine. 2011;18:1099-104.

[13] James GW, Daniela; Hastie, Trevor; Tibshirani, Robert. An Introduction to Statistical Learning with Applications in R: Springer; 2013.

[14] Vapnik VN. An Over View of Statistical Learning Theory. IEEE TRANSACTIONS ON NEURAL NETWORKS. 1999;10.
